# Newborn infants’ hair cortisol levels reflect chronic maternal stress during pregnancy

**DOI:** 10.1371/journal.pone.0200279

**Published:** 2018-07-06

**Authors:** Borja Romero-Gonzalez, Rafael A. Caparros-Gonzalez, Raquel Gonzalez-Perez, Pilar Delgado-Puertas, Maria Isabel Peralta-Ramirez

**Affiliations:** 1 Brain, Mind and Behavior Research Center (CIMCYC), Faculty of Psychology, University of Granada, Granada, Spain; 2 Department of Personality, Assessment and Psychological Treatment, University of Granada, Granada, Spain; 3 Gynecology and Obstetrics Department, Hospital de Poniente, El Ejido, Spain; 4 Department of Pharmacology, CIBERehd, School of Pharmacy, Instituto de Investigación Biosanitaria ibs.GRANADA, University of Granada, Granada, Spain; Universita Cattolica del Sacro Cuore Sede di Roma, ITALY

## Abstract

Cortisol obtained from hair samples represents a retrospective biomarker of chronic stress experienced by the subject in previous months. Although hair cortisol levels have been used to study the relationship between maternal and neonatal stress levels in primates, this has not yet been performed in humans using a longitudinal design and focusing specifically on this association. Therefore, the objective of this study was to determine whether a relationship existed between maternal psychological stress and hair cortisol levels during pregnancy and postpartum, and neonatal hair cortisol levels. The sample consisted of 80 pregnant women and their 80 newborn infants. We conducted a longitudinal assessment of hair cortisol levels, psychological stress, anxiety, and depression in the three trimesters of pregnancy and postpartum. After childbirth, neonatal hair cortisol levels were also measured. We found that maternal hair cortisol levels in the first trimester negatively predicted neonatal hair cortisol levels. Perceived stress in the third trimester of pregnancy also predicted lower neonatal cortisol, whereas pregnancy-specific stress in the same trimester had a positive relation with neonatal cortisol. Cortisol is essential for embryonic and fetal development; consequently, if fetal synthesis of cortisol is affected by high maternal cortisol levels, such development could be impaired.

## Introduction

High levels of prenatal stress can have negative consequences for maternal, fetal, and infant health [1–3]. The various methods used to assess stress during pregnancy and postpartum [4,5] include the administration of psychological questionnaires and the measurement of hair cortisol, which is a non-invasive biological method for obtaining retrospective information on chronic stress [6–8]. The relationship between psychological stress and cortisol levels in pregnant women is inconsistent, as it seems to depend on the matrix of the cortisol sample [9]. For example, there seems to be a relationship between salivary cortisol and anxiety and depression [10]. However, several authors have proved that there is no association between hair cortisol levels and self-reported symptoms of prenatal distress or perceived stress [11, 12].

High hair cortisol levels during pregnancy have been associated with an increased risk of miscarriage, premature birth, and low weight at birth [13, 14]; however, no conclusive results have been reported concerning the relationship between maternal cortisol and neonatal cortisol at birth. Primate studies have found higher maternal hair cortisol levels in the latter stages of pregnancy, and a negative relationship between this and birth weight [15, 16]. They have also reported lower hair cortisol levels in infant monkeys born to mothers exposed to stress during pregnancy [17].

In humans, only a few studies have analyzed maternal stress levels in relation to those of their newborn infants [18–20], and a positive relationship has been found between maternal and infant hair cortisol at 6, 9, and 12 months after birth [18, 20]. These studies were carried out by taking hair cortisol samples after birth, without taking into account the pregnancy [20]. Only one study has compared hair cortisol levels in mothers and premature babies immediately after birth, but no relationship was found [19]. However, no study on humans has taken the entire pregnancy into consideration.

Therefore, the objective of the present study was to analyze the relationship between maternal psychological stress and hair cortisol levels throughout the three trimesters of pregnancy and postpartum, and neonatal hair cortisol levels.

## Materials and methods

### Participants

**Fig 1:**
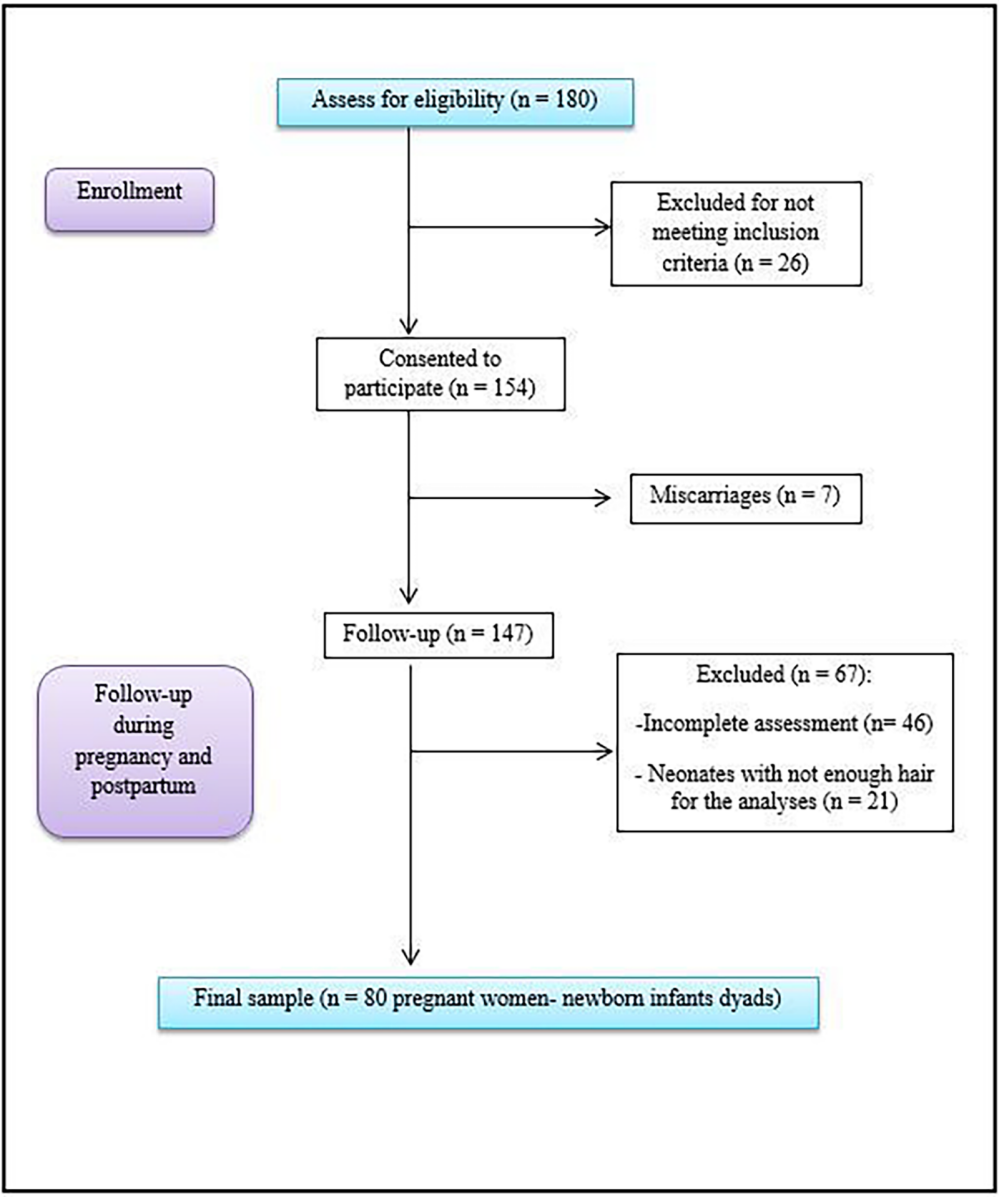
Participant follow-up flow diagram.

A total of 154 pregnant women from the Góngora Health Center (Granada), Roquetas de Mar Health Center (Almeria), and the Poniente Hospital in El Ejido (Almeria) signed an informed consent form. Seven had a miscarriage and 46 were excluded from the study for failure to complete all assessments. A further 21 were excluded because their newborn infants did not have sufficient hair for cortisol extraction. Hence, the final study sample comprised 80 pregnant women and 80 newborn infants (Fig 1). The women were longitudinally assessed in the first trimester of pregnancy (*M =* 11.48 weeks of gestation, *SD =* 3.72), the second trimester (*M =* 24.19 weeks of gestation, *SD =* 3.75), and the third trimester (*M =* 34.49 weeks of gestation, *SD =* 2.46). In addition, hair samples were collected from mothers and infants after birth (*M =* 15.79 days postpartum, *SD =* 9.78). Data were collected from April 2016 to July 2017.

As inclusion criteria, participants were over 18 years of age, had a good level of spoken and written Spanish, and their pregnancy was being monitored in the public health system. In addition, their infants had to have a minimum amount of 10 mg hair on their heads in order to take and analyze hair samples [21].

The exclusion criteria were having been administered corticosteroids during pregnancy, high risk pregnancies, or being diagnosed with any psychiatric condition or physical disease.

The protocol study “Influence of the hypothalamic-pituitary-adrenal axis on maternal and fetal health during pregnancy, childbirth, and the puerperium period” met the ethical standards established by the Declaration of Helsinki (revised in Fortaleza, Brazil, 2013) and was reviewed and approved by the Ethics Committee for Human Research at the University of Granada (reference number 881) and the Research Ethics Committee of the public health service in Granada and Almeria. As part of the protocol, the present study entitled “Newborn infants’ hair cortisol levels reflect chronic maternal stress during pregnancy” was also reviewed and approved by the abovementioned institutional review boards.

### Measures

#### Hair cortisol levels

Activation of the hypothalamic-pituitary-adrenal (HPA) axis was assessed by taking approximately 150 strands of hair from the posterior vertex of the head, close to the scalp [22]. Harvesting samples from this region of the head ensured less intra-assay variance in cortisol levels [23]. In addition, the maximum length of each sample was defined as 3 cm, in order to reflect cortisol levels over the previous 3 months independently of race, sex, or age variability in hair growth rates [7, 24, 25]. Assuming an average growth rate of 1 cm/month, a 3 cm segment would contain cortisol that had been deposited over approximately the previous 3 months [26]. The hair samples were wrapped in a piece of aluminum foil to protect them from light and humidity, and were then stored in an envelope at room temperature until being sent to the Faculty of Pharmacy at the University of Granada for analysis.

Prior to analysis, each hair sample was washed twice in isopropanol, weighed, and then ground to a fine powder using a ball mill (Bullet Blender Storm, Swedesboro NJ) in order to break up the hair protein matrix and increase the surface area for extraction. Cortisol was extracted in HPLC-grade methanol by incubating the sample for 72 hours at room temperature in the dark with constant inversion using a rotator. After incubation, a vacuum evaporator (Centrivac, Heraeus, Hanau, Germany) was used to evaporate the supernatant until completely dry. Then, the extract was reconstituted in 150 ul of phosphate buffered saline at a pH of 8.0. After that, the sample was frozen at -20°C until subsequent analysis [21, 26, 27].

The cortisol was measured using the Salivary ELISA Cortisol kit with the reagent provided following the manufacturer’s instructions (Alpco Diagnostics, Windham, NH). The Salivary ELISA Cortisol kit is a validated method to assess hair cortisol levels, is highly positively correlated with liquid chromatograph–mass spectrometry (LC–MS/MS) [28], and has been successfully used during pregnancy [4]. The sensitivity of the ELISA Cortisol kit is 1.0 ng/ml as reported by the manufacturer, and its cross-reactivity is as follows: Prednisolone 13.6%, corticosterone 7.6%, deoxycorticosterone 7.2%, progesterone 7.2%, cortisone 6.2%, deoxycortisol 5.6%, prednisone 5.6%, and dexamethasone 1.6%. No cross-reactions were detected with DHEAS or tetrahydrocortisone.

The intra- and inter-assay variations were analyzed on internal quality controls used for routine salivary cortisol measurement, measured in duplicate in eight consecutive assays. The intra-assay coefficients of variance (CV) were 2.7% at 10.7 ng/ml and 4.3% at 43.9 ng/ml. The inter-assay CVs were 4.4% and 6.3%, respectively.

#### Psychological assessment

Demographic information was collected from the Pregnancy Health Document [29], which is the official health record for pregnant women and their newborns.

For psychological assessment, psychological stress was evaluated using the Spanish version of the *Perceived Stress Scale* (PSS) [30, 31], which is a 14-item scale to evaluate the perception of general stress during the preceding month. Each of the 14 items is scored using a 5-point Likert scale (0 = never, 1 = almost never, 2 = once in a while, 3 = often, 4 = very often). The Cronbach’s alpha reliability coefficient for the Spanish version is α = 0.81 [31].

In addition, the Spanish version of the *Prenatal Distress Questionnaire* (PDQ) [32, 33] was used to assess pregnancy-specific stress. This instrument assesses specific worries and concerns that pregnant women experience regarding medical problems, physical symptoms, body changes, childbirth, relationships, labor, and the baby’s health using a 12-item scale scored with a 5-point Likert scale from 0 (none at all) to 4 (extremely). The Cronbach’s alpha reliability coefficient for the Spanish version is *α* = 0.71 [33].

Psychopathological symptoms were assessed using the Spanish version of the *Symptom Checklist 90 Revised* (SCL-90-R) [34, 35]. In order to facilitate participant assessment and due to the relation between cortisol levels and anxiety/depression, only the depression and anxiety sub-scales were used (depression sub-scale, Cronbach’s *α* = 0.88; anxiety sub-scale, Cronbach’s *α* = 0.83) [35].

#### Procedures

When the women attended their first antenatal appointment at 12 weeks’ gestation, the midwife informed them about the study. Those who agreed to participate were given an information sheet to read and signed an informed consent form. At this point, each participant was assigned an identification code to ensure anonymity throughout the study. Subsequently, the midwife distributed psychological questionnaires in paper format (PDQ, PSS, SCL-90-R), which the participants completed at home and submitted at their next appointment. At the same time, the midwife took hair samples in accordance with the established protocol [22]. This sequence was repeated in the second and third trimesters of pregnancy.

After birth, the midwife administered the psychological questionnaires to the mothers (PSS and SCL-90-R) and took a hair sample from each of them and their newborn infants. This evaluation was performed during the first postpartum appointment with the midwife.

#### Statistical analyses

First, we checked differences between included and excluded participants as regards sociodemographic and obstetric variables and stress levels. To this end, we performed Student’s t-tests for age and PDQ and PSS mean scores (quantitative variables) and Chi-square tests for the remaining variables, which were all categorical.

Second, given the main determinants of hair cortisol levels in adults and children [12, 36], variables such as maternal age, gestational age at birth, and birth weight were correlated with the outcome variable to determine potential confounding variables. In addition, two Student’s t-tests were performed to check differences between hair cortisol levels and maternal hair treatment (dyed or natural) and sex of the fetus (male or female).

A multicollinearity diagnosis was then performed in order to examine the associations between predictors, predicted variables, and covariates. A tolerance statistic higher than 0.3 and a variance inflation factor (VIF) ≤ 10 indicate a lack of multicollinearity [37].

To determine the relationship between maternal hair cortisol levels during the three trimesters of pregnancy and postpartum, and neonatal hair cortisol levels, we next conducted hierarchical linear regression analyses by the enter method. The dependent variable was newborn infants’ hair cortisol levels. In the first step, a potential confounding variable was included (maternal hair treatment). Then, maternal hair cortisol levels during pregnancy were included in step two of the model.

Similarly, in order to determine whether the maternal stress variables were related to neonatal cortisol levels, we performed a hierarchical linear regression analysis with neonatal hair cortisol levels as the dependent variable, and PDQ and PSS scores as independent variables. Maternal hair treatment was included in the model as a covariate.

Finally, a hierarchical linear regression analysis was conducted with neonatal hair cortisol levels as the dependent variable and maternal anxiety and depression scores during pregnancy as independent variables. Maternal hair treatment was included in the model as a covariate.

In line with recommendations for statistical analysis of hair cortisol level data, we conducted a natural logarithmic transformation (natural log; *ln* base e). All statistical analyses were performed using the Statistical Package for the Social Sciences 20.0 for Windows, version 8.1 (SPSS, Armonk, New York).

## Results

### Description of the sample

The sample consisted of 80 pregnant women aged between 23 and 44 years old (*M =* 32.17 years, *SD =* 4.06), and 80 newborn infants (56 boys and 24 girls). Table 1 shows the included and excluded participants’ main sociodemographic and obstetric variables. Forty-one out of 46 excluded participants provided sufficient information to be included in the analyses. There were no differences between groups.

**Table 1:** Sociodemographic and obstetric variables of the sample.

		Participants n = 80 M(SD)/n(%)	Excluded n = 41 M(SD)/n(%)	Test^a^	*p*
***Sociodemographic variables***					
Age		32.17(4.06)	32.80(5.00)	0.75	0.45
Nationality	Spanish	62(77%)	36(88%)	1.87	0.13
	Immigrant	18(23%)	5(12%)		
Marital status	Married/cohabitant	77(96%)	41(100%)	1.57	0.28
	Single/divorced/widow	3(4%)	-		
Level of education	Primary school	1(1%)	-		
	Secondary school	16(20%)	12(29%)	1.74	0.41
	University	63(79%)	29(71%)		
Employment status	Unemployed	12(15%)	9(22%)	1.74	0.41
	Working	68(85%)	32(78%)		
Hair	Natural	43(54%)	-	-	-
	Dyed	37(46%)	-		
Smoker	Yes	7(9%)	5(12%)	0.36	0.38
	No	73(91%)	36(88%)		
Alcohol	Yes	1(1%)	3(7%)	3.12	0.11
	No	79(99%)	38(93%)		
***Obstetric information***					
Primiparous	Yes	48(60%)	19(46%)	2.04	0.10
	No	32(40%)	22(54%)		
Wanted pregnancy	Yes	72(90%)	37(90%)	0.00	0.61
	No	8(10%)	4(10%)		
Pregnancy method	Spontaneous	73(91%)	37(90%)	1.72	0.42
	Fertility treatment	7(9%)	4(10%)		
Previous miscarriages	0	67(84%)	32(78%)	0.59	0.74
	1	10(12%)	7(17%)		
	≥ 2	3(4%)	2(5%)		
Previous children	0	50(63%)	23(56%)	1.34	0.71
	1	25(31%)	13(32%)		
	≥ 2	5(6%)	5(12%)		
Sex of the fetus	Male	56(70%)	-	-	-
	Female	24(30%)	-		

Note

^a^ Student’s t-test used for quantitative variables and Chi-square test for categorical variables.

Regarding stress levels, 34 out of 46 participants obtained scores for PDQ and PSS. There were no differences between groups for PDQ scores in the first trimester (*t =* 0.61; *p* = 0.54) or second trimester (*t =* 1.63; *p* = 0.11), or for PSS scores in the first trimester (*t =* -0.54; *p* = 0.58) or second trimester (*t =* -0.20; *p* = 0.83).

### Cortisol and potential confounding variables

No statistically significant correlations were observed between neonatal cortisol levels and maternal age (*r* = -0.09; *p* = 0.39), birth weight (*r* = -0.06; *p* = 0.59), or gestational age at birth (*r* = 0.04; *p* = 0.67). Neither were any statistically significant differences in neonatal cortisol levels detected according to sex of the fetus (*t* = -0.45; *p =* 0.65).

With regard to potential maternal confounders, the Student’s t-test only showed differences between groups in hair cortisol levels and maternal hair treatment (natural or dyed) in the first trimester (*t* = -2.30; *p* ≤ 0.05). Maternal age was not correlated with maternal hair cortisol levels (S1 Table). Consequently, maternal hair treatment was included in the analyses as a potential confounder.

### Hierarchical linear regression analyses for neonatal hair cortisol levels

We conducted a hierarchical linear regression by the enter method. The dependent variable was neonatal hair cortisol levels while the independent variables were maternal hair cortisol levels during pregnancy. In addition, a confounding variable was identified (maternal hair treatment) and this was included in the model (step 1).

Model 1, with only the confounding variable, explained less than 1% of the variance (*F* = 0.43; *R*^*2*^ = 0.00; *p* > 0.05), whereas model 2, which included confounding variables and maternal hair cortisol levels, explained 14% of the variance (*F* = 2.50; *R*^*2*^ = 0.14; *p* ≤ 0.05).

Maternal hair cortisol levels during the first trimester were the strongest predictor of hair cortisol levels in newborn infants (*β* = -0.24; *t* = -2.45; *p* ≤ 0.05) (Table 2).

**Table 2:** Hierarchical regression between maternal hair cortisol levels and newborn infants’ cortisol levels.

			Model 1			Model 2		
	*M (*SD)	*M (*SD)^a^	β	SE B	*t*	β	SE B	*t*
**Maternal hair treatment**			-0.13	0.21	-0.66	-0.06	0.21	-0.31
**Cortisol T1**	529,80(653,74)	5.78(1.04)				-0.24	0.10	-2.45*
**Cortisol T2**	422,23(510,99)	5.76(0.70)				-0.11	0.15	-0.74
**Cortisol T3**	448,25(681,63)	5.76(0.77)				-0.24	0.13	-1.78
**Cortisol T4**	735,81(1300,86)	6.00(0.97)				0.16	0.10	1.51
**R**^**2**^				0.00			0.14	
**F for changes in R**^**2**^				0.43			3.01*	

*Note =* *Significant at *p ≤* 0.05

^*a*^Mean and standard deviation of logarithmic transformation of cortisol levels

Dependent variable: Hair cortisol levels in newborn infants

T1 = trimester 1; T2 = trimester 2; T3 = trimester 3; T4 = Postpartum period.

Diagnosis of multicollinearity showed adequate tolerance (confounding variables > 0.60; independent variables > 0.80) and VIF < 10.

### Hierarchical linear regression analyses for psychological stress and newborn infants’ hair cortisol levels

We conducted a hierarchical linear regression by the enter method with newborn hair cortisol levels as the dependent variable and PDQ and PSS scores as the independent variables. In addition, maternal hair treatment was included in the model (step 1) as a confounding variable.

Model 1, which included maternal hair treatment, explained less than 1% of the variance (*F* = 0.43; *R*^*2*^ = 0.00; *p* > 0.05), whereas model 2 explained 22% of the variance (*F* = 2.54; *R*^*2*^ = 0.22; *p* < 0.05). The second model showed two variables that predicted newborn infants’ cortisol levels: PDQ scores in the third trimester (*β* = 0.08; *t* = 2.88; *p* < 0.01) and PSS scores in the same trimester (β = -0.28; *t* = -3.13; *p* < 0.01). The results are shown in Table 3.

**Table 3:** Multiple linear regression using psychological stress to predict neonatal hair cortisol levels.

	*M (SD)*	Model 1			Model 2		
		β	SE B	*t*	β	SE B	*t*
**Maternal hair treatment**		-0.13	0.21	-0.66	-0.20	0.20	-1.02
**PDQ T1**	14.23(3.84)				-0.01	0.02	-0.41
**PDQ T2**	12.88(3.49)				-0.04	0.03	-1.33
**PDQ T3**	13.10(3.90)				0.08	0.02	2.88**
**PSS T1**	26.88(1.10)				-0.10	0.09	-1.12
**PSS T2**	26.83(1.15)				0.00	0.09	0.00
**PSS T3**	27.08(1.20)				-0.28	0.09	-3.13**
**PSS T4**	26.90(1.20)				0.06	0.08	0.71
**R**^**2**^			0.00			0.22	
**F for changes in R**^**2**^			0.43			2.84*	

*Note =* **Significant at *p ≤* 0.01

Dependent variable: Hair cortisol levels in newborn infants

PDQ = Prenatal Distress Questionnaire; PSS = Perceived Stress Scale; T1 = trimester 1; T2 = trimester 2; T3 = trimester 3; T4 = Postpartum period.

The tolerance statistic for variables was higher than 0.70, and VIF was also satisfactory (VIF < 10).

With respect to anxiety and depression, the scores did not predict neonatal hair cortisol levels. Pearson’s correlation between maternal and neonatal cortisol levels and psychological stress, anxiety, and depression are shown in S2 Table.

## Discussion

The objective of this study was to analyze the relationship between psychological stress and hair cortisol levels in mothers during pregnancy and postpartum, and neonatal hair cortisol levels, based on a longitudinal assessment. The results showed that higher maternal hair cortisol levels in the first trimester of pregnancy predicted lower neonatal hair cortisol. In addition, pregnancy-specific stress and perceived stress were also predictors of neonatal hair cortisol.

First, we found that high hair cortisol levels in the first trimester predicted lower hair cortisol levels in newborn infants. This negative relationship is in agreement with the only other study conducted with a similar objective, on rhesus monkeys, which found lower hair cortisol levels in infants of mothers who were highly stressed during the first trimester of pregnancy [17]. It is known that high levels of stress during the third trimester affect the long-term development of the newborn infant [38]; however, our results seem to indicate that maternal cortisol affects fetal cortisol from the first trimester. The effect of timing and type of prenatal stress exposure has been related to different outcomes in the offspring [38, 39]; in this respect, our study shows an important time-specific relation between biological measures of stress, such as cortisol, and fetal programming. A possible explanation could be that during pregnancy, maternal cortisol crosses the placenta to the fetus, since the latter cannot produce it independently until later stages of gestation [40]. Maternal cortisol stimulates secretion of the corticotrophin release hormone (CRH) in the fetus, and in turn, CRH secretion stimulates cortisol synthesis [41, 42]. Hence, excessively high levels of cortisol crossing the placenta in the early stages of fetal formation probably alter fetal programming of the HPA axis, preventing correct cortisol production in the fetus [43].

Special attention should be paid to this phenomenon due to its implications for development, since low cortisol levels at birth require the administration of glucocorticoids to ensure the extrauterine life of the infant [44]. Furthermore, it is well known that the effect of prenatal stress on child development and infant stress regulation is explained by fetal programming of the HPA [42, 45]. Therefore, ensuring high neonatal cortisol levels could help promote good lung function and thus prevent the development of later diseases [46–48].

Similarly, the scores for perceived stress in the third trimester of pregnancy were negatively associated with neonatal cortisol levels, whereas pregnancy-specific stress in the third trimester was positively associated with neonatal cortisol levels. Although the relationship between these measures remains unknown, similar results have been reported when cortisol was measured in the umbilical cord of newborn infants, whereby those whose mothers had experienced more stress during pregnancy showed lower cortisol levels [49]. As our data indicate, there was no association between perceived and pregnancy-specific stress in our sample, and the trends in correlations with maternal and neonatal hair cortisol differed according to time and type of stress. It is important to note that perceived stress and pregnancy-specific stress are not the same measures of stress: Hence, the effects each could exert on infants are different [50]. Our results show that the effects of pregnancy-specific stress are different to those of perceived stress, as other authors have previously reported [4, 51].

As study limitations, a high number of participants were lost due to the longitudinal design and inclusion and exclusion criteria of the study. Another limitation is generalizability of the sample, since almost the entire sample lived with an intimate partner and there were no multiple pregnancies, which is not representative of the general population. Thus, future research should strive to include single mothers and those who have multiple pregnancies.

Nonetheless, the longitudinal design of this study and the simultaneous assessment of hair cortisol levels and psychological status have shed valuable light on relationships that have not previously been studied in humans, revealing a negative association between maternal hair cortisol in the first trimester and neonatal hair cortisol levels. Future research could include long-term follow-up of newborn infants in order to elucidate the implications of cortisol levels for subsequent neurodevelopment.

It is important to determine the effect of maternal stress on the fetus and identify critical periods such as the first trimester, as has been demonstrated in the present study. Since stress during pregnancy has negative consequences for the fetus and newborn infant, the main challenge is to ensure early identification and prevention of high stress levels [52–54]. Although there is evidence that postnatal care can help neonates at risk [55, 56], prenatal assessment of stress levels and maintenance of optimal levels remain important. Collecting data on cortisol levels and psychological well-being may help early detection of problems that could affect the intrauterine relationship between mother and fetus, which is fundamental for the future health of both.

## Conclusions

This is the first study to analyze the relationship between maternal hair cortisol during pregnancy and neonatal hair cortisol. The analysis was complemented by psychological assessment of the mother throughout pregnancy.

This enabled us to determine that maternal hair cortisol in the first trimester exerts a negative influence on neonatal hair cortisol levels. These findings have important clinical implications, since the detection of vulnerable trimesters during pregnancy could contribute to minimize the adverse effects of psychological and physiological stress during pregnancy for the newborn infant and subsequent child.

## Supporting information

S1 Table Pearson correlation and Student’s t-tests between potential confounding variables for neonatal and maternal cortisol levels. (DOCX)

S2 Table Pearson correlation between psychological and biological measures. (DOCX)
