# Supplementary material for: Newborn infants’ hair cortisol levels reflect chronic maternal stress during pregnancy
Source: PLoS One. 2018 Jul 6;13(7):e0200279. doi: 10.1371/journal.pone.0200279 (PMC6034834; doi:10.1371/journal.pone.0200279)
Supplement: S1 Table — (DOCX) [file pone.0200279.s001.docx]

**S1 Table.** Pearson correlation and Student’s t-tests between potential confounding variables for neonatal and maternal cortisol levels

|  |  | | X(DT) | t/*r* | *p* |
| --- | --- | --- | --- | --- | --- |
| **Neonatal cortisol levels** |  |  |  |  |  |
| Sex |  | Male | 7.52(0.93) | -0.45 | 0.65 |
|  |  | Female | 7.62(0.94) |  |  |
|  |  |  |  |  |  |
| Maternal age |  |  |  | -0.09 | 0.39 |
| Birth weight |  |  |  | -0.06 | 0.59 |
| Gestational age at birth |  |  |  | 0.04 | 0.67 |
|  |  |  |  |  |  |
| **Maternal cortisol levels** |  |  |  |  |  |
| Trimester 1 | Maternal hair treatment | Natural | 6.02(1.02) | -2.30 | **0.02^*^** |
|  |  | Dyed | 5.50(1.00) |  |  |
|  | Maternal age |  |  | -0.14 | 0.21 |
| Trimester 2 | Maternal hair treatment | Natural | 5.73(0.75) | 0.36 | 0.71 |
|  |  | Dyed | 5.79(0.65) |  |  |
|  | Maternal age |  |  | 0.07 | 0.53 |
| Trimester 3 | Maternal hair treatment | Natural | 5.77(0.91) | -0.12 | 0.89 |
|  |  | Dyed | 5.74(0.59) |  |  |
|  | Maternal age |  |  | 0.01 | 0.92 |
| Postpartum | Maternal hair treatment | Natural | 6.15(1.02) | -1.51 | 0.13 |
|  |  | Dyed | 5.82(0.90) |  |  |
|  | Maternal age |  |  | -0.02 | 0.82 |

*Note:* *Significant at p ≤ 0.05
