# Supplementary material for: Newborn infants’ hair cortisol levels reflect chronic maternal stress during pregnancy
Source: PLoS One. 2018 Jul 6;13(7):e0200279. doi: 10.1371/journal.pone.0200279 (PMC6034834; doi:10.1371/journal.pone.0200279)
Supplement: S2 Table — (DOCX) [file pone.0200279.s002.docx]

**S2 Table. Pearson correlation between psychological and biological measures.**

|  | Cortisol T1 | Cortisol T2 | Cortisol T3 | Cortisol T4 | Cortisol NB |
| --- | --- | --- | --- | --- | --- |
| PDQ T1 | -0.10 | -0.08 | 0.00 | -0.17 | 0.05 |
| PDQ T2 | 0.14 | -0.19 | 0.04 | **-0.24^*^** | -0.12 |
| PDQ T3 | 0.04 | 0.05 | 0.08 | 0.08 | 0.17 |
|  |  |  |  |  |  |
| PSS T1 | -0.02 | 0.11 | -0.00 | 0.05 | -0.14 |
| PSS T2 | 0.08 | -0.01 | 0.03 | **-0.24^*^** | -0.07 |
| PSS T3 | -0.24 | -0.09 | 0.11 | -0.06 | **-0.32^**^** |
| PSS T4 | 0.14 | -0.07 | -0.04 | -0.13 | -0.02 |
|  |  |  |  |  |  |
| ANX T1 | -0.01 | 0.02 | 0.02 | **-0.23^*^** | -0.02 |
| ANX T2 | 0.08 | 0.01 | 0.09 | -0.17 | -0.08 |
| ANX T3 | 0.05 | 0.03 | 0.21 | 0.06 | -0.04 |
| ANX T4 | 0.03 | -0.10 | 0.02 | -0.03 | -0.02 |
|  |  |  |  |  |  |
| DEP T1 | -0.05 | 0.08 | 0.10 | -0.14 | -0.14 |
| DEP T2 | 0.11 | 0.06 | 0.07 | -0.09 | -0.06 |
| DEP T3 | 0.15 | 0.06 | 0.14 | 0.00 | -0.06 |
| DEP T4 | -0.03 | -0.02 | 0.05 | 0.04 | -0.04 |

*Note:* Significant at ^*^*p* ≤ .05 and ^**^*p* ≤ .01

T1 = Trimester 1; T2 = Trimester 2; T3= Trimester 3; T4 = Postpartum; NB = Newborn infants; PDQ = Prenatal Distress Questionnaire; PSS = Perceived Stress Scale; ANX = Anxiety sub-scale SCL-90-R; DEP = Depression sub-scale SCL-90-R
